# The Queen's Jubilee Hospital

**Published:** 1905-02-25

**Authors:** 


### THE QUEEN'S JUBILEE HOSPITAL.

### A NEW COMMITTEE OF MANAGEMENT WANTED.

Last week we announced that the matron of the Queen's Jubilee Hospital and the whole of the medical staff, with the exception of Mr. Benham and the dentist, had sent in their resignations; to-day, on another page, we publish a letter which we have received from the late members of the staff, together with two resolutions which they adopted and presented to the board of management when tendering their resignations. We do not think that anybody who has been familiar with the internal economy and working arrangements at the Queen's Jubilee Hospital during past years will be in the least surprised at the course which the matron and medical staff have found it necessary to take. Having used their best endeavours to remedy the numerous defects in the administration of the hospital, and to bring about several desirable reforms, without avail, there was but one resource left, if they were to avoid implication in the abuses which they felt were rife. That their complaints were well founded is unquestionable, and it is extraordinary that the hospital authorities have systematically ignored them. But it is hard to believe that those at present responsible for the management of the institution fully appreciate the duties entrusted to them.

The wholesale withdrawal of the professional staff of a hospital is a very unusual event, and is sufficiently grave to demand a searching investigation. The proper course for the hospital authorities to pursue in such a case would be to institute an inquiry before accepting the resignations. But so little desire for enlightenment is possessed by the management of the Queen's Jubilee Hospital that the resignations have been accepted without any inquiry or material discussion, and the posts are already being advertised as vacant. We suspect, however, that it will not be an easy matter to obtain suitable candidates for the vacancies. Seeing that here have been four different matrons in the short space of two years, it would seem as though the position were not an enviable one; and if the report be true that the matron is not permitted to control the discipline and behaviour of her nurses we can understand why the appointment is subject to such frequent changes. It will, we think, be even more difficult to find properly qualified gentlemen to take charge of the various medical departments, for honourable and self-respecting members of the profession will be chary of hazarding their reputations by the venture. Indeed, it would appear that the authorities of the Queen's Jubilee Hospital already feel this difficulty, for in their current advertisements they omit to make any mention of the rule, which formerly held good, and which stipulated that no one should be appointed to the consulting staff who did not possess those adequate qualifications which are considered indispensable at every hospital claiming the confidence of the public. It cannot be expected that any self-respecting person will voluntarily associate himself with the hospital, whether as a member of the staff, or as a subscriber, or in any other capacity, so long as the present inefficiency is permitted to continue. An entirely new and enlightened committee of management is not only desirable, it is absolutely necessary if the Queen's Jubilee Hospital is to have the confidence of the public and so obtain the funds necessary for its existence as a charitable institution; for it is wholly unendowed. Unless some earnest endeavour is made forthwith to save the situation in this way the Queen's Jubilee Hospital must inevitably sink from discredit out of existence altogether.

